# Urinary angiotensinogen antedates the development of stage 3 CKD in patients with type 1 diabetes mellitus

**DOI:** 10.14814/phy2.14242

**Published:** 2019-10-11

**Authors:** Sheeba Ba Aqeel, Minghao Ye, Jan Wysocki, Alejandro Sanchez, Ahmed Khattab, Enrique Lores, Alfred Rademaker, Xiaoyu Gao, Ionut Bebu, Robert G. Nelson, Mark Molitch, Daniel Batlle, For the Diabetes Control, Complications Trial (DCCT)/Epidemiology of Diabetes Interventions, Complications (EDIC) Study

**Affiliations:** ^1^ Northwestern University Feinberg School of Medicine Chicago Illinois; ^2^ George Washington University Rockville Maryland; ^3^ National Institute of Diabetes and Digestive and Kidney Diseases Phoenix Arizona

**Keywords:** biomarkers, chronic kidney disease, diabetes, hypertension, renin angiotensin system, urinary angiotensinogen

## Abstract

We examined if urinary angiotensinogen (uAOG), a marker of intrarenal renin‐angiotensin system activity, antedates stage 3 chronic kidney disease (CKD) using samples from participants in the Diabetes Control and Complications Trial (DCCT) and later in the Epidemiology of Diabetes Intervention and Complications (EDIC) trial. In a nested case–control design, cases were matched at the outcome visit (eGFR less than 60, 21‐59 mL/min per 1.73 m^2^) on age, gender, and diabetes duration, with controls: eGFR (95, 75‐119, mL/min per 1.73 m^2^.) Additionally, in an exploratory analysis progressive renal decline (PRD), defined as eGFR loss >3.5 mL/min per 1.73m^2^/year, was evaluated using only data from EDIC because no progressions were observed during DCCT. At the EDIC visit, which antedated the GFR outcome visit by 2 years (range 1–7years) the median uAOG/creatinine was markedly higher in cases than in controls (13.9 vs. 3.8 ng/mg *P* = 0.003) whereas at the DCCT visit, which antedated the GFR outcome by 17 to 20 years it was not (2.75 vs. 3.16 ng/mg, respectively). The Odds Ratio for uAOG and CKD stage 3 development was significant after adjusting for eGFR, HbA1c, and systolic blood pressure 1.82 (1.00–3.29) but no longer significant when Albumin Excretion Ratio (AER) was included 1.21 (0.65–2.24).In the PRD analysis, uAOG/creatinine was sixfold higher in participants who experienced PRD than in those who did not (26 vs. 4.0 ng/mg, *P* = 0.003). The Odds Ratio for uAOG and PRD was significant after adjusting for eGFR, HbA1c, and systolic blood pressure 2.48 (1.46–4.22) but no longer significant when AER was included 1.32 (0.76–2.30). In people with type1 diabetes, a robust increase in uAOG antedates the development of stage 3 CKD but is not superior to AER in predicting this renal outcome. Increased uAOG moreover is associated with PRD, an index of progression to End Stage Kidney Disease (ESKD).

## Introduction

Diabetic Kidney Disease (DKD) is an important microvascular complication of both type 1 and type 2 diabetes (Parving, 2001; Batlle, 2003; Perkins et al., 2007; Molitch et al., 2010; Rosolowsky et al., 2011; Campion et al., 2017; Umanath and Lewis, 2018). The renin angiotensin system (RAS) is a pathway importantly involved in chronic kidney disease progression and a current target for standard of care therapies to slow down the progression of CKD (Brenner et al., 2001; Zeeuw et al., 2004; Lewis et al., 2001; Ruggenenti and Remuzzi, 2019). Urinary angiotensinogen is the parent compound of all the angiotensin peptides, as it provides the substrate for the cascade of events that lead to formation of Angiotensin II (Ingelfinger et al., 1990; Anderson et al., 1993; Kobori et al., 2002; Liu et al., 2008; Nakano et al., 2012; Matsusaka et al., 2012; Wysocki et al., 2017). In animal studies, urinary AOG is highly correlated with intrarenal AOG and angiotensin II levels therefore suggesting that it may be a marker of intrarenal RAS activity (Ingelfinger et al., 1990; Anderson et al., 1993; Kobori et al., 2002; Liu et al., 2008; Wysocki et al., 2017). A corollary of this is that elevated uAOG levels can inform on the status of the kidney RAS and possibly aid in the decision to initiate RAS blocker therapy to slow down the progression to CKD.

There is evidence from cross‐sectional studies that uAOG is increased in patients with DKD, (Yamamoto et al., 2007; Kobori et al., 2008; Mills et al., 2012; Afkarian et al., 2014; Wysocki et al., 2017; Juretzko et al., 2017) but longitudinal data from patients with either type 1 or type 2 diabetes, to our knowledge, are lacking. Accordingly, at what point kidney RAS overactivity develops during the evolution of DKD is unknown. Early blockade of the RAS in normoalbuminuric normotensive patients with type 1 diabetes did not slow progression to nephropathy (Mauer et al., 2009; Mann et al., 2009). This could reflect that activation of the kidney RAS in type 1 diabetes is not an early event with the implication that early initiation of RAS blockers may not be necessary prior to the clinical appearance of CKD. From the aforementioned reasons we reasoned that information on uAOG, a marker of the kidney RAS activity, during the evolution to CKD stage 3 in type 1 diabetes would be helpful to understand the pathophysiology of the disease and help in deciding the timing for initiation of RAS blocker therapy.

In this report, we examined whether elevated uAOG levels antedated the development of early CKD (stage 3) in patients with type 1 diabetes over an extended follow‐up. The long duration of this unique study, provided us with the opportunity to examine uAOG on two separate occasions many years apart and both prior the development of the study outcome, GFR <60 mL/min per 1.73m^2^. One sample was available during a DCCT visit almost 20 years prior to the outcome and the other sample during an EDIC visit a few years prior to the GFR outcome, In addition to this primary study aim, we evaluated, in an exploratory analysis, if uAOG is associated with progressive renal decline, a strong index of future ESRD development (Krolewski and Bonventre, 2012; Krolewski et al., 2014, 2014; Krolewski, 2015; Skupien et al., 2016; Krolewski et al., 2017). Our studies were done using urine bio samples stored from participants in the Diabetes Control and Complications Trial (DCCT)/Epidemiology of Diabetes Intervention and Complications (EDIC) (The Diabetes Control and Complications Trial Research Group, 1993; The Diabetes Control and Complications Trial Research Group, 1994; Boer et al., 2011).

## Materials and methods

The DCCT was a multicenter randomized clinical trial to test the effects of conventional versus intensive control of blood glucose on the complications of diabetes. 1,441 volunteers, ages 13 to 39, with type 1 diabetes of 1‐15 years duration were enrolled at 29 medical centers in the United States and Canada (The Diabetes Control and Complications Trial Research Group, 1993; The Diabetes Control and Complications Trial Research Group, 1994). Following completion of the DCCT, all participants were encouraged to receive intensive treatment and they were returned to their health care providers for ongoing diabetes care. They were also invited to join the EDIC study, an observational ongoing extension of the DCCT, and 1375 (96% of the surviving cohort) agreed to do so (The Diabetes Control and Complications Trial Research Group, 1994). This study using DCCT/EDIC historical samples was considered exempt by the Northwestern University IRB.

### Study design

#### Primary analysis

We designed a nested case–control study of participants in the DCCT/EDIC study (Ernster, 1994; Boer et al., 2011) to test the association of uAOG with DKD, defined by reaching stage 3 CKD. Cases were defined as reaching sustained (in 2 successive measurements) eGFR values of <60 mL/min per 1.73 m^2^ through EDIC year 18. Controls had persistent normal kidney function defined by both eGFR >60 mL/min per 1.73 m^2^ and AER <30 mg/24 hr at study entry1In one of the controls who met these criteria, however, there was one isolated AER measurement of 46mg/24hr at the DCCT study visit; we kept this control despite the misclassification because all other early samples throughout the DCCT/EDIC were in the normal range in this subject. and through the outcome visit on which the corresponding case participant developed eGFR <60mL/min per 1.73 m^2^. At the outcome visit, controls were matched to each case (one or more controls per case) based on gender, age, and duration of diabetes (within 3‐years of each other) as well as baseline eGFR. Each case was matched to one or more controls who were free of event at the event time of the case (Robins et al., 1986). Based on urine sample availability, we had 34 cases (from 34 participants) and 51 matched controls (from 39 participants) because in a nested case–control study design controls can be matched to more than one case. In all instances two urine samples were used, one from the earliest visit in DCCT and the other from the earliest visit in EDIC that the sample was made available to us. Urinary AOG and creatinine were assayed in these two samples; study visit in DCCT (17‐20 years prior to outcome) and study visit in EDIC (1‐7 years prior to outcome). Urine samples were provided from the DCCT/EDIC bio sample repository at the University of Minnesota.

#### Secondary post hoc analysis: Progressive renal decline

**Figure 1:**
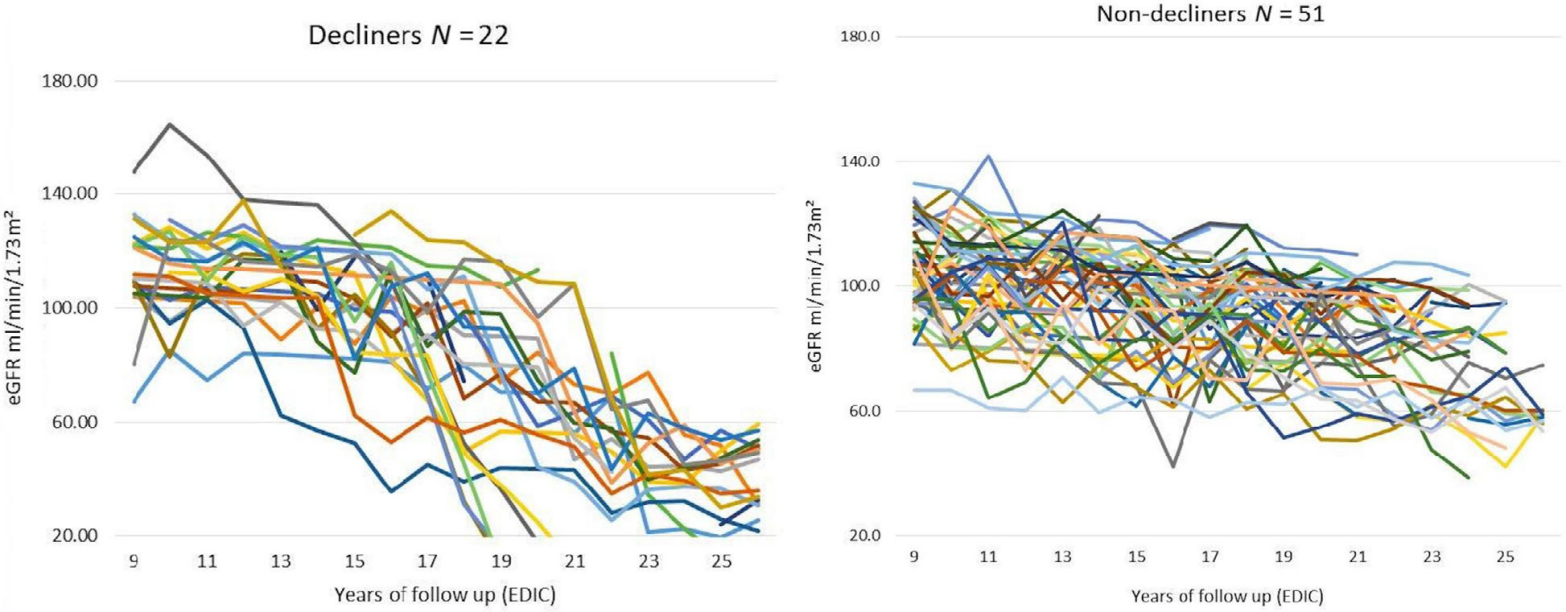
Left figure shows eGFR trajectories in n = 22 of 73 subjects in EDIC (which started at year 9 following the first 9 years of DCCT) who developed progressive renal decline of >3.5 mL/min per 1.73m^2^/year. Right figure shows eGFR trajectories in *N* = 51 of 73 subjects that maintained eGFR at <3.5 mL/min per 1.73 m^2^/year.

The progressive renal decline concept is a model developed by the Joslin Clinic group to assess the progression of people with Type 1 diabetes to ESKD. In their studies involving a large number of patients with type 1 diabetes it was found that a fall in eGFR of more than 3.5 mL/min strongly predicts the development of ESKD. We therefore investigated the association between uAOG and Progressive Renal Decline (PRD), defined as eGFR loss of >3.5 mL/min per 1.73 m^2^/year (Krolewski and Bonventre, 2012; Skupien et al., 2012; Krolewski et al., 2014, 2014; Krolewski, 2015; Krolewski et al., 2017). None of the cases or controls had a fall in eGFR >3.5 mL/min per 1.73 m^2^/year during the DCCT; therefore, the association of uAOG with PRD was examined based on EDIC data only. For this exploratory analysis, uAOG was compared in the 22 subjects who developed PRD, that is, eGFR loss of >3.5 mL/min per 1.73 m^2^/year (referred to as decliners) and the 51 subjects who did not (referred to as nondecliners) (Fig. 1A,B). All 22 decliners came from the 34 cases described in the primary analysis whereas the 51 nondecliners came from the 39 controls and 12 of the 34 cases who did not meet the definition of PDR. In a sensitivity analysis, comparisons were also made by excluding these 12 cases leaving the number of nondecliners to a total of only 39.

#### Urinary Angiotensinogen and creatinine Measurements

Aliquots of urine were collected using standardized procedures and stored at <−70°C at the participating centers and later stored at the DCCT repository in Minnesota at −90°C. Available urine samples were transferred to our laboratory at Northwestern University where uAOG was measured using a Human Total Angiotensinogen Assay Kit from IBL (Japan).

Since the DCCT/EDIC samples arrived at the Northwestern University laboratory, urine sample thawing and freezing was kept to the minimum (1 to 2 cycles if repeat measurements were required). Urinary AOG measurements were minimally affected after two or five freeze–thaw cycles (96% and 92% of 1 freeze–thaw reference, respectively). The AOG kit employed a solid‐phase sandwich ELISA whereby uAOG was captured by one AOG antibody, which was coated onto the micro‐titer plate. Horseradish peroxidase‐conjugated AOG antibody was then added and tetramethylbenzidine (TMB) was used as a chromogen. The reaction was stopped by the addition of 1N H_2_SO_4_ and the color intensity, which was proportional to the AOG concentration, was read using a 450‐nm filter. The measurement range of the assay is 0.31–20 ng/mL (6.0–384.6 pmol/L). Urinary creatinine was also measured in our laboratory using Parameter^TM^ creatinine assay kit (R&D Systems). The inter‐assay variability for creatinine in the urinary samples was 13.2% with an inter‐assay correlation coefficient of *R* ^2^ = 0.9305. The intra‐assay coefficient of variation for creatinine was 0.8% (*n* = 40 measurements). The inter‐assay coefficient of variation for uAOG was 5.7%, and the intra‐assay coefficient of variation was 4.4% (*n* = 100 measurements).

Urinary creatinine was also measured in our laboratory using Parameter^TM^ creatinine assay kit (R&D Systems). uAOG was divided by the creatinine concentration and expressed as ng/mg creatinine (Hsu et al., 2015). The effect of frequent freeze–thaw was tested recently in urine DCCT samples which underwent one, two or five consecutive freeze–thaw cycles in liquid nitrogen prior to uAOG quantification (Afkarian et al., 2014). uAOG was minimally affected after two or five freeze–thaw cycles (96% and 92% of 1 freeze–thaw reference, respectively. Urinary creatinine was also measured in our laboratory using Parameter^TM^ creatinine assay kit (R&D Systems). uAOG was divided by the creatinine concentration and expressed as ng/mg creatinine (Hsu et al., 2015). Data on 24 hour urinary albumin excretion and eGFR, calculated using the CKD‐EPI formula, was provided to us by the DCCT/EPIC study group.

### Statistical analysis

Since the values of uAOG and AER were not normally distributed, we transformed them into logarithmic values using natural logarithm (Bilous et al., 2009). Conditional logistic regression was used to determine the association between case–control status and eGFR as well as log urinary AOG measured at the earliest DCCT and EDIC visits prior to development of Stage 3 CKD. The association between log uAOG and risk of CKD was adjusted for eGFR, HbA1c, blood pressure, use of RAS blockers and other anti‐hypertensive medicines and log AER. Repeated measures analysis of variance with Tukey adjusted post hoc tests was used to compare eGFR between DCCT and EDIC visits for both cases and controls.

For the analysis of PRD, we used serial measurements of eGFR and extracted the linear component of each trajectory as a simple slope (from the first EDIC year (follow‐up year 9) through EDIC year 18 (follow‐up year 25) for each subject), to characterize the distribution of rates of eGFR decline for each subject, as described by Krolewski et al. (2014); Krolewski (2015); Skupien et al. (2016); Krolewski et al, (2017). Wilcoxon test was used to test the differences between unadjusted medians in the decliners and nondecliners. In exploratory analyses, multivariable logistic regression models were used to assess the association between log uAOG (measured at the EDIC study visit only) and PRD. The association between log uAOG and PRD was adjusted for eGFR, HbA1c, blood pressure and log AER, to examine if they were mediators in the relation between log uAOG and PRD. These analyses included the calculation of receiver operating curves and areas under the ROC curve. Odds ratios and 95% confidence intervals are given. A *P*‐value of <0.05 was considered statistically significant.

## Results

### Studies during DCCT

**Figure 2:**
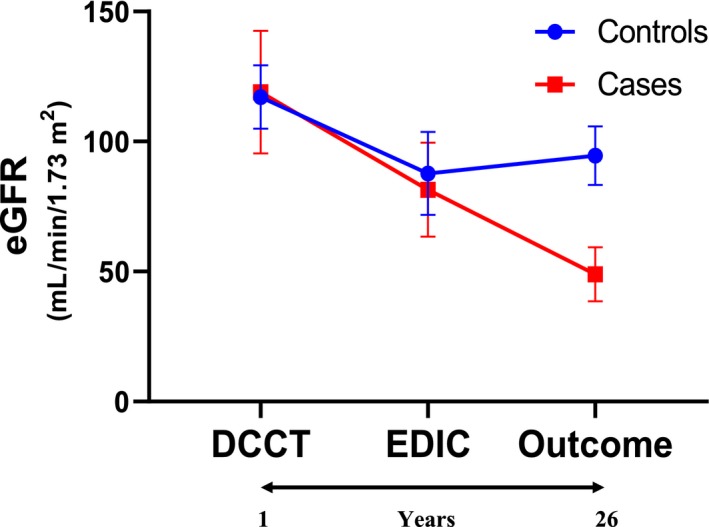
Changes in eGFR (ml/min per 1.73 m^2^) over time expressed as mean ± SE at three time points: DCCT, EDIC and outcome visits. DCCT visit was at year 1 for cases and controls whereas the EDIC visit was at year 18 and year 16 for cases and controls, respectively, (see Table 1 for range and results for further description). Matching of controls with cases was done at the outcome visit when eGFR had fallen below 60 mL/min per 1.73 m^2^ in cases but not in controls. Therefore, at the outcome visit eGFR was markedly different by study design. By Tukey's multiple comparisons post hoc there was a significant decline in both cases and controls from the DCCT to the EDIC visit (adjusted *P* < 0.0001).

The characteristics of cases and controls during the DCCT study visit are shown in Table 1. The median of this visit was in year 1 of the DCCT study but varied both in cases and controls depending on sample availability (see range). This early DCCT study visit preceded the eGFR study outcome (which occurred always during EDIC follow‐up) by 17 to 20 years as shown in Figure 2 that depicts the evolution of eGFR throughout DCCT, and EDIC visits prior to the GFR outcome visit.The eGFR was not significantly different between cases and controls (Table 1).

**Table 1:** Characteristics of cases and controls at DCCT and EDIC study visits.

Clinical parameters	DCCT			EDIC		
	Cases N = 34	Controls N = 51	*P*‐value*	Cases N = 34	Controls N = 51	*P*‐value*
EDIC/DCCT year visit**	1 (0–3)	1 (0–7)	0.47	18 (8–26)	16 (9–24)	0.003
Age (years)	35 (14–41)	36 (14–44)	0.85	51 (25–62)	51 (25–57)	0.016
Males %	74	68	0.51	74	68	0.51
Disease duration (months)	61 (25–184)	70 (26–210)	0.29	276 (108–408)	240 (132–408)	0.028
HbA1C (%)	8.7 (6.0–15)	8.0 (5–11)	0.23	8.3 (5.5–12.7)	8.0 (6.3–10.7)	0.026
SBP (mmHg)	114 (90–138)	116 (90–134)	0.86	139 (104–173)	133 (102–155)	0.043
DBP (mmHg)	74 (60–90)	74 (58–88)	0.87	83 (56–102)	80 (60–100)	0.63
eGFR (ml/min per 1.73 m^2^)	119 (81–207)	113 (88–148)	0.86	76 ( 60–119)	91 (62–120)	0.02
Intensive treatment (%)	9 (35%)	11 (32%)	0.8	13 (38%)	21 (41%)	0.82
Standard treatment (%)	17 (65%)	23 (68%)	0.8	21 (62%)	30 (59%)	0.82
RAS Blockers (%)	0	0		68	31	0.002
Anti‐hypertensives (%)	0	0		76	33	<0.001

Even though the group statistics are similar or identical, the p‐values are significant in some cases because comparisons are made within matched sets for the conditional logistic regression

* *P*‐value as obtained per conditional regression analysis.

** The DCCT year visit is the earliest visit in DCCT when the study sample was available.

Cases and controls were similar with respect to age, gender, and diabetes duration. The HbA1c was higher in cases but not significantly different than in the controls. Both systolic blood pressure (SBP) and diastolic blood pressure (DBP) were not significantly different in the two groups Allocation to intensive or standard care during DCCT was not significantly different between cases and controls. None of the cases or controls were taking RAS blockers or other anti‐hypertensives drugs during this visit or throughout the DCCT.

The median uAOG/creatinine ratio in cases was not significantly different from controls (Table 2). The median AER in cases and controls were essentially the same (median 10.0 vs. 10.1 mg/24 hr, respectively, *P* = 0.61) (Table 2). Only in two cases and in one control the AER was above 30 mg/ 24 hr.

**Table 2:** Urine AOG/ creatinine ratio (ng/mg) and AER (mg/24 hr) in the DCCT and EDIC study visits prior to outcome.

	Cases N = 34	Controls N = 51	*P*‐value*
**DCCT visit**. AOG/Creatinine Ratio (ng/mg) (Median and range)	2.75 (0.38–30)	3.16 (0.53–26)	0.67
**EDIC visit**. AOG/Creatinine ratio (ng/mg) (Median and range)	13.9 (0.96–989)	3.83 (0.44–228)	0.003
**DCCT visit**. AER (mg/24hr) (Median and range)	10.0 (1.4–99.7)	10.1 (4–46)	0.61
**EDIC visit**. AER (mg/24hr) (Median and range)	86 (4.3–7357)	9 (3–20)	0.01

Urine AOG/ creatinine (ng/mg) was measured prior to outcome (eGFR <60 mL/min per 1.73m^2.^) in DCCT visit (range 17‐20 years prior to outcome) and in EDIC visit (range 1–7 years prior to outcome).

* Reflects the *P*‐value obtained as per conditional regression analysis.

### Studies during EDIC

The characteristics of cases and controls during the EDIC study visit are shown in Table 1. The median year study visit was 18 with a range of 8‐26 years in cases and 16 with a range of 9‐24 years in controls. HbA1c and SBP were significantly higher in cases than in controls.

In both cases and controls, eGFR had fallen significantly at the EDIC visit as compared with the DCCT visit (Figure 2). In each of the cases and controls the eGFR was higher than 60 mL/min per 1.73m^2^ by study design but despite of this matching criteria the eGFR in this visit was significantly lower in cases than in controls when assessed by conditional regression analysis to be consistent with the analysis of all the other parameters (Table 1).

During the EDIC visit, 18% of cases had developed microalbuminuria, 41% had developed macroalbuminuria, and 41% remained normoalbuminuric. The controls, by contrast, had not developed micro or macroalbuminuria as per study design. During the EDIC visit, many patients were treated based on standard of care and because many had developed micro or macroalbuminuria, the proportion of patients taking RAS blockers or other anti‐hypertensive drugs was much higher in cases than among controls.

The median uAOG/creatinine ratio at the EDIC study visit was about threefold higher in the cases as compared with the controls (13.9 vs. 3.8 ng/mg, respectively, *P* = 0.003) (Table 2). By conditional logistic regression, higher log transformed uAOG/creatinine was associated with the development of Stage 3 CKD: OR (95% CI) 2.05 (1.27–3.31), *P* = 0.003 (Table 3). This persisted after simultaneous adjustment for eGFR, HBA1c, SBP, and DBP but not after adjusting for log AER (Table 3).

**Table 3:** Odds ratio and 95% CI (confidence interval) for urinary AOG and CKD development (EDIC visit).

	Odds ratio	95% CI
AOG/creatinine (ng/mg)	2.05	1.27–3.31
Adjusted for eGFR, HBA1c, SBP and DBP	1.82	1.00–3.29
Adjusted for eGFR, HBA1c, SBP and DBP and AER (mg/24 hr)	1.21	0.65–2.24

The ODDS ratio was significant for AOG/ Creatinine (ng/mg) alone (upper row) and adjusted for eGFR, HBA1c, SBP and DBP ( middle row) but not when AER is included (lower row).

**Figure 3:**
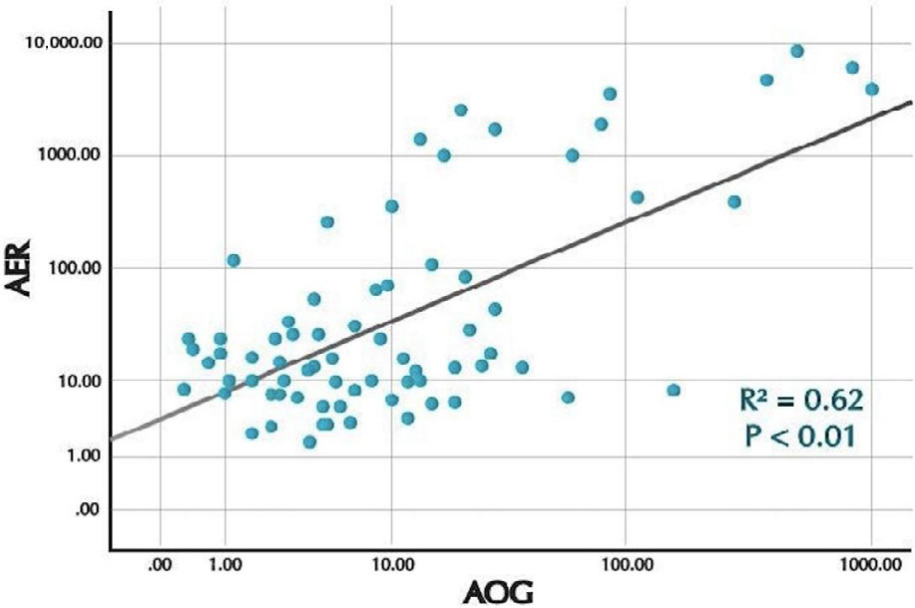
Scatterplots and correlation between log urine AOG and log AER (*R* ^2^ = 0.62, *P* < 0.01) during the DCCT and EDIC study visits combined. AER = Albumin excretion rate (mg/24 hr). AOG = Urine angiotensinogen, AOG/creatinine ratio (ng/mg).

This higher AOG level antedated the development of eGFR <60mL/min per 1.73 m^2^ in each of the cases. The median time between the measurement of the high uAOG/creatinine level and development of stage 3 CKD, that is, a decrease in eGFR below 60mL/min per 1.73 m^2^, was 2 years (range 1–7 years). A positive correlation between uAOG and AER was found by linear regression using data from DCCT and EDIC study visits combined (*R* ^2^ = 0.62) (Figure 3).

### Studies during EDIC on progressive renal decline (PRD)

This exploratory analysis was aimed at examining the possible association of increased uAOG/creatinine with PRD, and index of future ESRD development (Krolewski, 2015; Skupien et al., 2016; Krolewski et al., 2017). This analysis involves data from all the participants (total 73) studied during the EDIC part of the study only (the 34 cases and the 39 controls). Of the 73 patients studied, 22 had a slope of eGFR >−3.5 mL/min per 1.73 m^2^/year that met the definition of PRD (Decliners) (Figure 1A) while the remaining 51 maintained a more steady slope of eGFR (nondecliners) (Figure 1B).

As noted in the methods, all the 22 decliners were cases in the analysis of the primary study outcome (eGFR <60 mL/min per 1.73 m^2^), whereas the 51 nondecliners originated from 12 cases and 39 controls. Among the 22 decliners, the mean rate of decline was 6.3mL/min per 1.73 m^2^/year and median slope was −5.01 mL/min per 1.73 m^2^/year. Among the 51 nondecliners the mean rate of decline was −1.4 mL/min per 1.73 m^2^/year and the median slope was −1.27 mL/min per 1.73 m^2^/year.

The characteristics of the 22 decliners and 51 nondecliners at the study visit in EDIC are given in Table 4. At this visit the two groups were similar with respect to age, gender, and disease duration and the eGFR was above 60 mL/min per 1.73 m^2^ in both groups and not significantly different from each other (85 vs. 91 mL/min per 1.73 m^2^, *P* = 0.4). The median HbA1c and AER were significantly higher in the decliners than in the nondecliners. Neither systolic nor diastolic BP was significantly different between the two groups. Allocation in intensive versus standard insulin was not significantly different between the two groups.

**Table 4:** Characteristics of decliners and nondecliners at the EDIC study visit.

Clinical parameters	Decliners; N = 22	Nondecliners; N = 51	*P*‐value*
EDIC year visit	18 (8–26)	15 (9–24)	0.12
Age (years)	47 (25–62)	51 (28–61)	0.05
Males %	45	61	0.99
Disease duration (months)	261 (108–408)	260 (132–408)	0.66
HbA1C (%)	9.1 (5.5–12.7)	8.1 (6–11)	0.03
SBP (mmHg)	140 (104–173)	134 (102–155)	0.09
DBP (mmHg)	83 (56–102)	81 (60–100)	0.81
Intensive treatment (%)	10 (45%)	19 (37%)	0.6
Standard treatment(%)	12 (55%)	32 (63%)	0.6
eGFR (ml/min per 1.73 m^2^) Median and range	85 (61–119)	91 (61–120)	0.47
AER (mg/24 hr) Median and range	391 (4.7–357)	10 (1–951)	<0.001
AOG/creat (ng/mg) Median and range	26 (1–989)	4.0 (0.4–228)	0.003

* *P* value as per Wilcoxon test.

The median uAOG/creatinine ratio was sixfold higher in the decliners compared with the nondecliners, (26.1 vs. 4.0 ng/mg, *P* = 0.003) (Table 4). A sensitivity analysis was done with only 39 of the 51 participants who were considered as nondecliners by excluding 12 participants who were controls in the primary analysis. In the sensitivity analysis, AOG/creatinine (ng/mg) was similarly more than sixfold higher in the 22 decliners: 26.1 (1.0–989 ng/mg) than in these 39 nondecliners: 3.9 (0.4–228 ng/mg); *P* < 0.001. AER was also higher in the decliners than in t those 39 nondecliners: 391 (4.3–7357) and 10.1 (2.9–36) mg/24hr, respectively; *P* < 0.001.

Log transformed uAOG/creatinine was associated with the development of PRD: OR (95% CI) 2.23 (1.45–3.43). This persisted after simultaneous adjustment for eGFR, HbA1c and SBD and DBP. After adjusting for log AER, however, the difference was no longer significant (Table 5).

**Table 5:** Odds ratio and 95% CI (confidence interval) for urinary AOG and progressive renal decline (EDIC visit).

	Odds ratio	95% CI
AOG/creatinine (ng/mg)	2.23	1.45–3.43
Adjusted for eGFR, HBA1c, SBP, and DBP	2.48	1.46–4.22
Adjusted for eGFR, HBA1c, SBP, and DBP and AER (mg/24 hr)	1.32	0.76–2.30

The Odds ratio was significant for AOG/ Creatinine (ng/mg) alone (upper row) and adjusted for eGFR, HBA1c, SBP and DBP ( middle row) but not when AER is included ( lower row).

**Figure 4:**
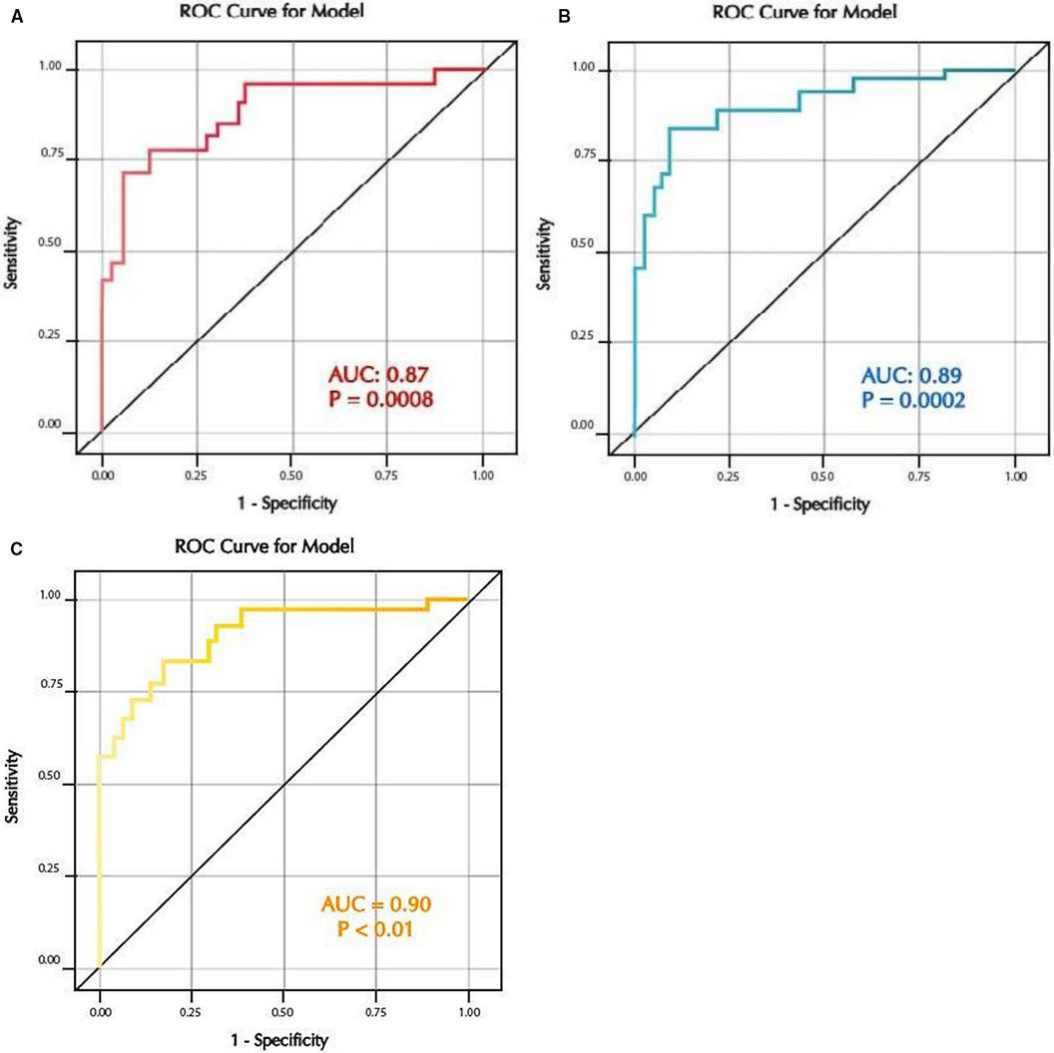
(A) Receiver Operating Curve for log uAOG/creat after adjusting for eGFR, HbA1C, SBP and DBP. AUC = 0.87, *P* = 0.0008. (B) Receiver Operating Curve for log AER after adjusting for eGFR, HbA1C, SBP and DBP. AUC = 0.89, *P* = 0.0002. (C) Receiver Operating Curve for log AER + log AOG after adjusting for eGFR, HbA1C, SBP and DBP. AUC = 0.90, *P *< 0.01.

The receiver operating curve (ROC) characteristics of the 22 decliners and the 51 nondecliners are shown in Figure 4. The area under the curve (AUC) for log transformed uAOG/creatinine unadjusted for any covariates was 0.79, *P* < 0.001. The AUC for log transformed uAOG/creatinine after adjusting for eGFR, HbA1c and blood pressure was 0.87, *P* < 0.001 (Figure 4A). This was similar to the AUC of 0.89 for log AER after adjusting for eGFR, HbA1c and blood pressure, *P* < 0.001 (Figure 4B). Using the method of DeLong et al., there is no significant difference in the two AUCs of 0.87 for log AOG and 0.89 for log AER (*P* = 0.65)(DeLong et al., 1988). Thus, both AOG and AER gave similar values for PRD based on ROC analysis. After concurrent adjustment of variables (eGFR, Hb1c and systolic BP) the AUC for the combined log AOG and log AER was 0.90, *P* < 0.01 (Figure 4C).

## Discussion

This study shows that in patients with type 1 diabetes uAOG expressed as AOG/ creatinine ratio is elevated before the development of stage 3 CKD (eGFR <60 mL/min per 1.73 m^2^). By conditional logistic regression moreover, a higher log transformed uAOG/urine creatinine ratio was associated with the development of CKD stage 3 and this association persisted after combined adjustment for eGFR, HBA1c, and blood pressure but not AER (Table 3). AER was already increased at the time that uAOG was measured and at this time uAOG was found to be increased as well. Although increased uAOG does not predict development of CKD stage 3 better than increased AER it provides information regarding the pathophysiology of RAS within the kidney over time and insight into the progression of CKD.

The higher uAOG level in cases was documented a median of 2 years (range 1–7 years) before the eGFR fell below <60 mL/min per 1.73 m^2^.During the DCCT visit, 17 to 20 years prior to the primary outcome, by contrast, uAOG was not different from controls (Table 2). At this remote time point, AER was within the normal range and there were also no differences between cases and controls in this established biomarker of kidney disease. Biomarkers are useful if they can improve our understanding of disease pathophysiology, accurately stratify patients based on disease risk or stage, help in identifying the initiation of kidney disease and predict response to specific therapies (Tummalapalli et al., 2016; Campion et al., 2017). In this study, eGFR declined relatively rapidly from the time that uAOG was found to be elevated during the EDIC phase (Figure 2). Since uAOG is considered a marker of activation of RAS within the kidney, (Urushihara et al., 2010; Kobori and Navar, 2011) the observed rapid eGFR decline from the time that uAOG was found elevated may reflect that an overactive RAS is involved in the progression to CKD. By extrapolation, RAS blockade would seem more effective when such RAS overactivity can be documented by a relatively easy measurement of a key RAS component of this system such as urinary AOG.

RAS blockers are known to reduce the levels of urinary and kidney AOG by suppressing angiotensin II (Ang II), a positive regulator of AOG (Kobori et al., 1979; Ba Aqeel et al., 2017). Of note, elevated uAOG was found despite the fact that RAS blockers were used far more frequently in cases than in the controls during EDIC (Table 1). Accordingly, if the cases had not been taking RAS blockers, one could assume that the increase in levels of uAOG/creatinine would have been even higher than the already threefold increase observed in median AOG/creatinine levels. This observation suggests potential value of uAOG/creatinine for timing of initiation of RAS therapy. In other words, when uAOG levels are elevated there would be a stronger rationale to use these agents to slow down the progression of CKD. This information may be even more relevant now that other therapies, namely SGLT‐2 inhibitors, are being introduced for the management and prevention of diabetic kidney disease (Ingelfinger and Rosen, 2019). Of note, these agents activate the RAS owing to the volume depletion that results from sodium and glucose diuresis (Cherney et al., 2014). While clinical trials of renoprotection in diabetic kidney disease are currently being done in comparison with standard care, which includes treatment with RAS blockers, it seems reasonable to anticipate that the use of these now traditional agents will need to be more selective to avoid unnecessary pill burden, costs, and associated potential side effects.

In a secondary analysis, higher levels of uAOG/creatinine were associated with progressive renal decline, a strong index of progression to ESKD in patients with type 1 diabetes (Krolewski, 2015; Krolewski et al., 2017). This analysis, however, is only exploratory, as it involved the same nested cohort of cases and controls studied during the EDIC phase that was designed to examine the development of Stage 3 CKD as the primary outcome. Despite this limitation, the sixfold increase in levels of uAOG in decliners as compared with the nondecliners was quite robust. A strong association between increased uAOG/ creatinine and PRD, moreover, was independent of eGFR, HbA1c and blood pressure (Table 5). When adjusted for AER, however, the association was no longer significant. AOG and AER were similar in the AUC analysis for PRD (AUC 0.87 and 0.89, respectively) (Figure 4). The AUC analysis based on case control studies using matching on risk factors yields biased estimates and therefore caution is needed in their interpretation (Robins et al., 1986; Ernster, 1994). Notwithstanding the fact that this limitation applies to our exploratory analysis, the AUC for both AER and AOG show strong associations with PRD, a marker of ESKD development (Krolewski, 2015; Krolewski et al., 2017).

The almost identical values in the AUC for uAOG and AER may reflect, to a large extent, similarities in the renal handling of these proteins. Both albumin and AOG are proteins of similar molecular size such that both can be filtered (Nakano et al., 2012). Therefore, both can be excreted in increased amounts early in CKD even with very mild alterations in the glomerular filtration barrier. Consistent with this concept we found a positive, albeit not strong, correlation between uAOG and 24‐hour albumin excretion, *R* ^2^ = 0.62, *P* < 0.001 (Figure 3). The observed positive correlation would be concordant with the *pari passu* appearance in urine via glomerular filtration of both these proteins during the evolution of kidney disease in type 1 diabetes. In this regard, urine AOG could be viewed as a filtration marker much in the same way as urinary albumin. Even a very strong positive correlation between AER and uAOG, however, does not rule out increased formation of AOG intra‐renally. The two proteins may increase in parallel but for different reasons, that is, an increase in filtration in the case of albumin and both an increase in filtration and in intrarenal formation in the case of AOG. In cross‐sectional studies involving patients with CKD, an association between increased uAOG and reduced eGFR was found independently of AER (Mills et al., 2012; Juretzko et al., 2017). This would further support the concept that elevated uAOG is not solely the result of passage of AOG via altered glomerular permeability but also excretion of AOG that originates, in part, from local intra‐renal formation. From our data, however, we cannot rule out a major contribution from liver derived AOG that is filtered, and then, excreted in the urine.

Regardless of its origin, systemic or locally produced, excessive AOG may activate the kidney RAS locally. This is evident from studies of podocyte‐selective injury in transgenic mice that have shown that circulation‐derived AOG can activate kidney RAS when the glomerular filtration barrier is altered, indicating the dependency of kidney Ang II generation on filtered AOG (Matsusaka et al., 2014). An increase in kidney AOG could trigger RAS activation by providing the substrate for downstream formation of angiotensin peptides. AOG is the parent substrate of angiotensin peptides and its overproduction, particularly at the kidney level in diabetes (Lai et al., 1998; Kim et al., 2012), may further upregulate a cascade of reactions to form Ang II, thereby promoting kidney injury (Singh et al., 2005; Liu et al., 2008). Ang II is the key component of the RAS system that exerts pro‐inflammatory and pro‐fibrotic actions in the kidney leading to progression of renal injury (Siragy and Carey, 2010). That increased levels of uAOG reflect increased levels of Ang II in the kidneys is suggested from studies in rodent models of diabetes and in cross sectional clinical studies (Kobori et al., 2003; Yamamoto et al., 2007; Liu et al., 2008; Nishiyama et al., 2011; Wysocki et al., 2017). Moreover there is evidence that Ang II upregulates AOG synthesis both within the kidney and in the liver (Herrmann and Dzau, 1983; Kobori et al., 2004). This implies that a positive feedback may perpetuate Ang II formation in states of Ang II over activity which, in turn, exacerbates kidney injury.

A strength of our study is the prolonged follow‐up of the cases and controls from DCCT to EDIC to generate data prior to the outcome study visit (Figure 2). A limitation is that we did not have multiple urine samples available for serial measurements of AOG to examine when uAOG began to increase during the evolution of type 1 diabetes to stage 3 CKD. This information would also have allowed us to examine if the increase in uAOG always coincides or not with an increase in AER. At the EDIC study visit when uAOG was increased the AER was also increased and although the eGFR was above 60 mL/min per 1.73 m^2^ in both groups it was already slightly lower in cases than in controls. Of note, the within‐person variability of albuminuria is very high as recently shown by Waikar et al. (2018). It would therefore be of interest to examine the variability of AOG in longitudinal studies with multiple samples over time. If less variable than AER this would be an added value of uAOG as a more stable biomarker in addition to conveying information on the status of the kidney RAS.

In summary, our study is the first to show that elevated levels of uAOG antedate the development of stage 3 CKD in patients with type 1 diabetes. Our exploratory studies, moreover, show that elevated uAOG is associated with progressive renal decline, a strong marker of progression to ESRD. uAOG provides insight into the pathophysiology of progression of CKD in people with type 1 diabetes and may be useful in guiding the timingfor initiation of RAS blocker‐based therapies.

## Conflict of Interest

None.
